# Proctitis and Abdominal Aortic Aneurysm: Six Degrees of Separation

**DOI:** 10.7759/cureus.5129

**Published:** 2019-07-12

**Authors:** Sindhura Kolli, Simcha Weissman, Fahad Malik, Owen Chan, Mel A Ona

**Affiliations:** 1 Clinical Obesity Medicine, NYU Langone Health, New York, USA; 2 Internal Medicine, Hackensack University Medical Center, North Bergen, USA; 3 Internal Medicine, Richmond University Medical Center, Staten Island, USA; 4 Pathology, Pali Momi Medical Center, Affiliate of Hawaiʻi Pacific Health, Honolulu, USA; 5 Gastroenterology, Pali Momi Medical Center, Affiliate of Hawaiʻi Pacific Health, Honolulu, USA

**Keywords:** proctitis, cmv colitis, abdominal aortic aneurysm (aaa)

## Abstract

Cytomegalovirus (CMV) is an aggressive virus responsible for a considerable amount of case fatalities. In the overwhelming majority of cases, this affects only the immunocompromised. Herein, we present a 76-year-old immunocompetent female who presented with gastrointestinal bleeding found to have rectal ulceration secondary to CMV infection. This manuscript aims to raise awareness of a rare cause of rectal bleeding. Hopefully, as such, our case will also prevent long-standing inflammation from persisting in patients with CMV and prevent it from contributing to cardiovascular pathology as seen in our patient.

## Introduction

Cytomegalovirus (CMV) is an aggressive, potentially fatal virus that predominantly affects the immunocompromised. Thus, when it presents in the immunocompetent it can often be misleading. Also, CMV is known to affect the gastrointestinal tract by inducing colonic ulcerations [1]. The appearance of ulceration in the rectum is obscure and can lead to misdiagnosis [2].

Moreover, like other chronic disease processes, the delay in diagnosis can lead to series of clinical manifestations. With inflammation in particular, the manifestations can be harsh and appear in the form of cardiovascular demise. Herein, we present an immunocompetent 76-year-old female with a rapid history of cardiovascular disease found to have CMV-induced ulcers likely responsible for her cardiovascular phenomenon via inducing a long-standing state of systemic inflammation.

## Case presentation

**Figure 1:**
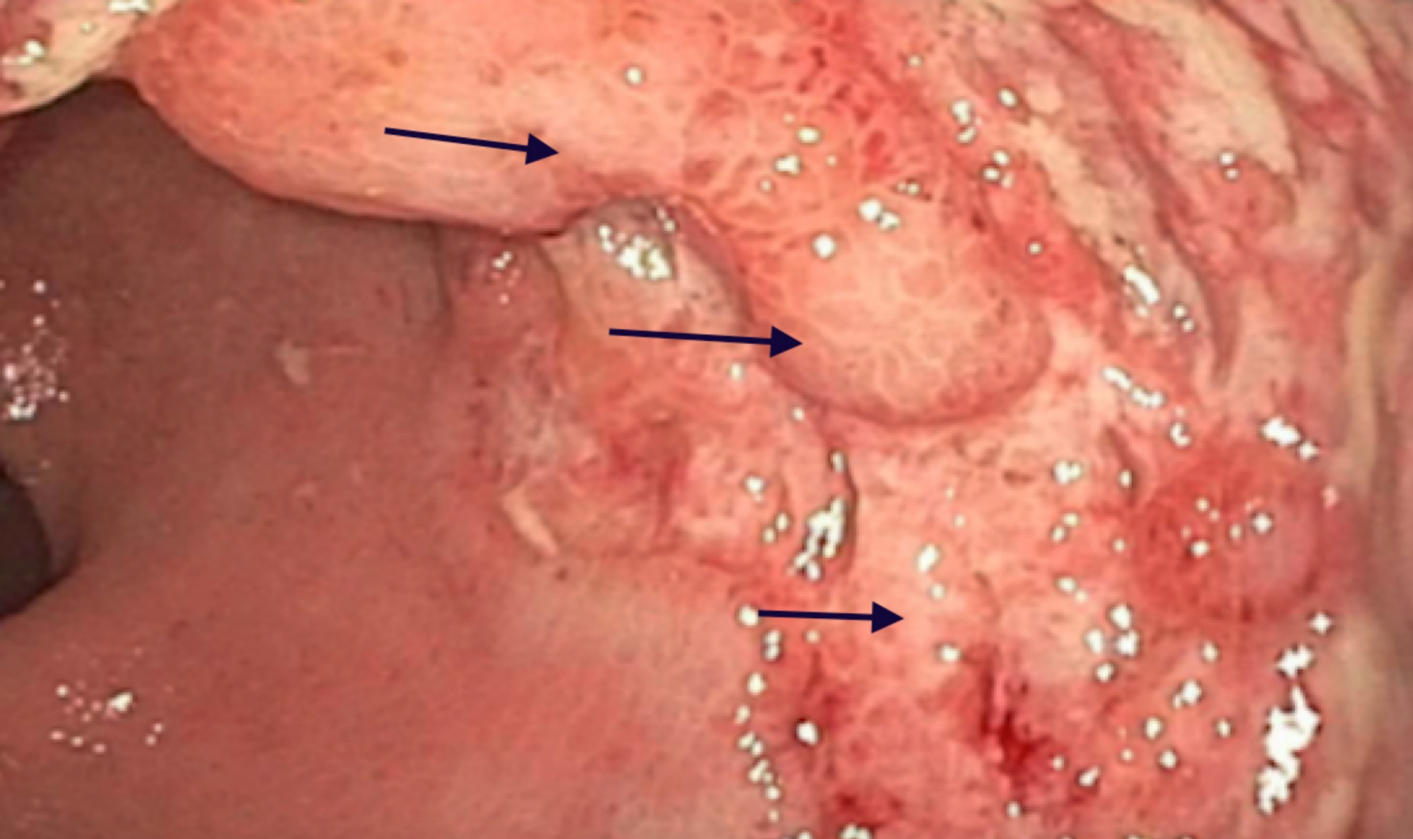
Colonoscopy revealing three centimeter (cm) rectal ulcer with heaped-up margins (black arrows)

A 76-year-old immunocompetent female presented to the emergency department (ED) complaining of chest pain and blood in the stool. She was recently discharged from the hospital status post endovascular repair for an abdominal aortic aneurysm (AAA) and hemodialysis for worsening renovascular disease. Troponin proteins were elevated, and electrocardiogram showed a non-ST-elevation myocardial infarction (NSTEMI), for which she underwent stent placement, and began anti-coagulation therapy. The lower endoscopic evaluation revealed a three-centimeter, clean-base, ulcerated non-obstructing mass containing heaped-up mucosal edges in the right/anterior portion of the rectum (Figure *1*).

**Figure 2:**
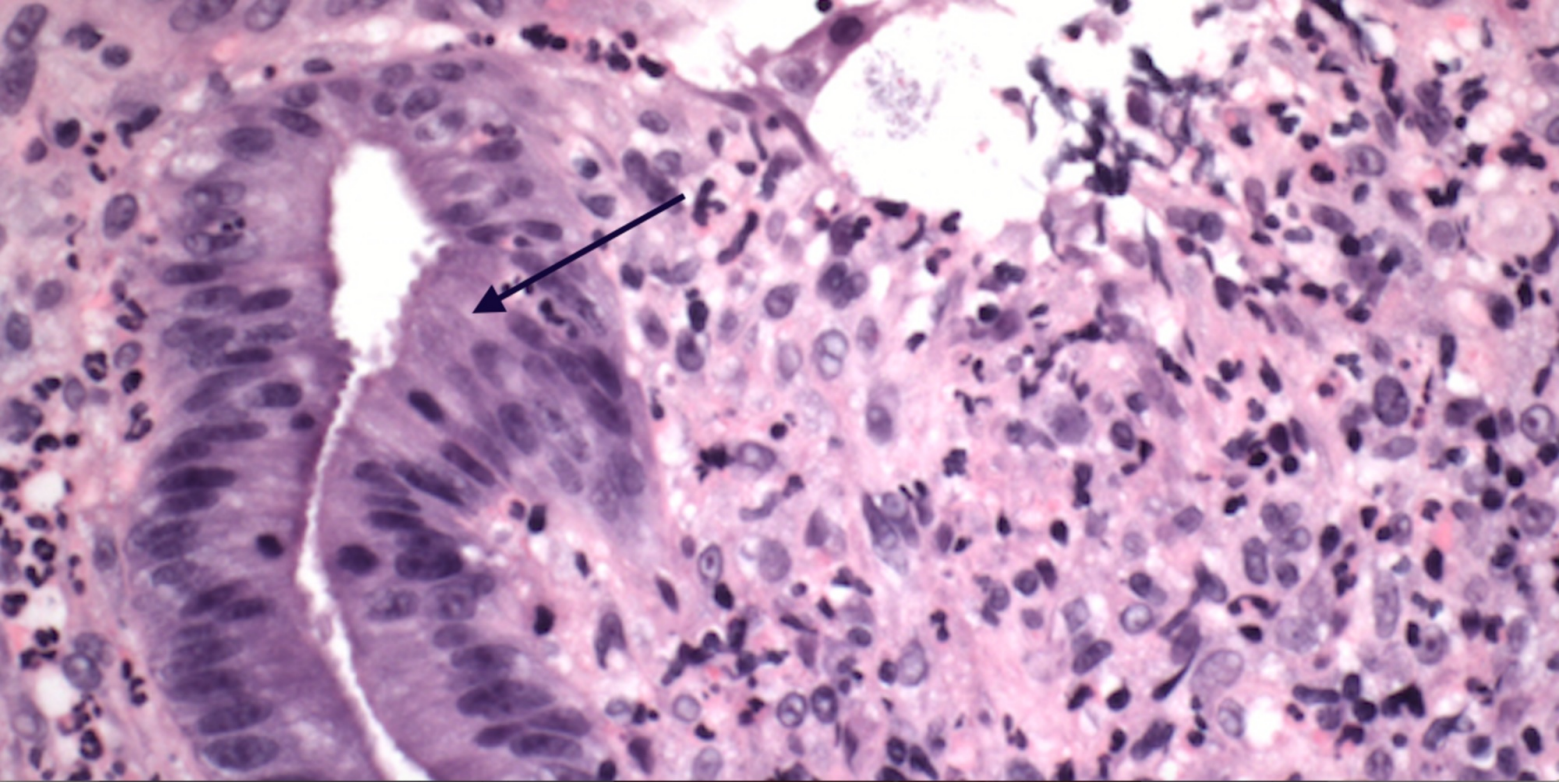
Light microscopy (hematoxylin and eosin, 400x) showing active proctitis and ulceration (black arrow)

The differential diagnosis included tumor, inflammatory bowel disease, ischemic proctopathy, infections, and stercoral ulceration. Hematoxylin and eosin (H&E) staining identified active proctitis with ulceration (Figure *2*). 

**Figure 3:**
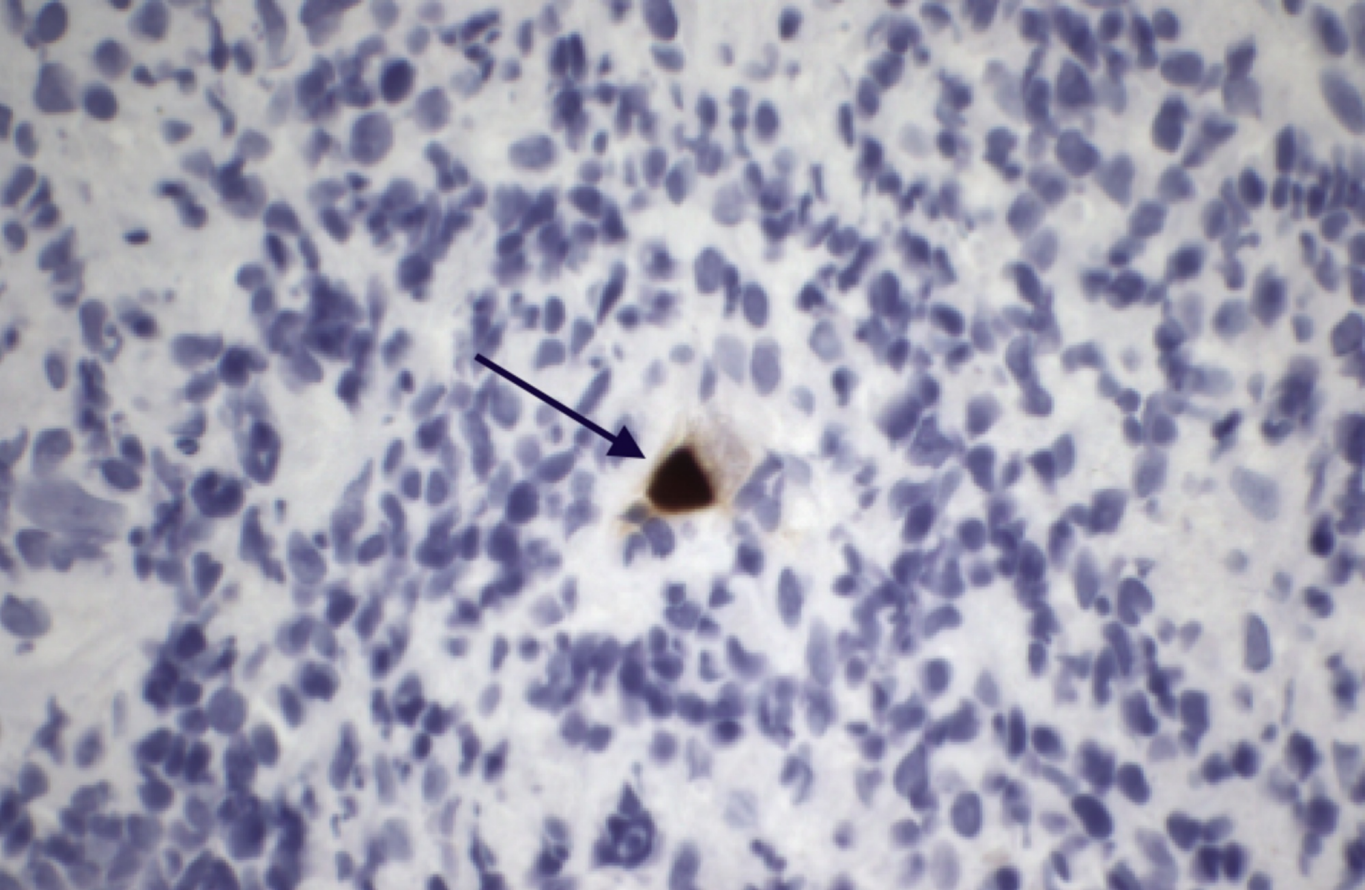
Immunostaining positive for cytomegalovirus (CMV) in the rectal ulcer with illustration of an ‘owl eye inclusion’ (black arrow)

Thereafter, immunostaining revealed CMV-infected cells, reaching a diagnosis of CMV infection without the need for seropositivity to be performed (Figure *3*). 

She began intravenous ganciclovir and within a few days she had resolution of symptoms. She was discharged in stable condition with instructions to follow up outpatient.

## Discussion

CMV-a double-stranded deoxyribonucleic acid (DNA) virus-is a prominent member of the herpes virus family. The prevalence of CMV infection in the general adult population is approximately 70% [3, 4]. CMV occurs via the excretion of bodily fluids and is transmission by close personal contact [5].

In the large majority of hosts, infection is usually asymptomatic [4]. Thus, usually only in the immunocompromised patient do the dangerous disease manifestations of CMV occur. CMV infection has a predilection for the lower gastrointestinal tract and primarily manifests as ulcerations along the colonic mucosa rarely involving the rectal region [3]. Our case illustrates the need to include CMV in the differential amongst the immunocompetent population especially in patients with comorbidities such as advanced kidney disease. Moreover, our case shows that ulcerations may reach as far down as the rectum and possibly contribute to disease throughout the intestine. 

Attention to diagnostic clues, such as ulcers with heaped-up margins, and extra measures to immunostain a biopsied sample to search for CMV-infected cells or ‘owl eye’ inclusion bodies on histology, can clinch the diagnosis. This becomes of upmost importance in unsuspected patients, those chronically infected with CMV, in order to prevent systemic inflammation reactions which may ultimately lead to serious cardiovascular sequelae as seen in our case. Once diagnosed, treatment involves anti-retroviral therapy with 2-3 weeks of intravenous ganciclovir.

Antiviral therapy is recommended for both immunocompromised and immunocompetent patients, as without this therapy CMV proctitis can otherwise result in poor patient outcomes [4, 6]. Additionally, it is important for clinicians to become knowledgeable as to the potential adverse reactions ganciclovir treatment can induce which include myelosuppression, hepatotoxicity, and nephrotoxicity [4, 5].

## Conclusions

In conclusion, clinicians must maintain a broad differential diagnosis in patients presenting with rectal ulcers of unknown identity. Attention to imaging and obtaining a strong patient history can help prevent fatal extra-disease sequelae. Although generally a disease of the immunocompromised, CMV can exist in the immunocompetent patient as illustrated in our patient.
